# Implementing teleophthalmology services to improve cost-effectiveness of the national eye care system

**DOI:** 10.1038/s41433-024-03156-4

**Published:** 2024-06-04

**Authors:** E. P. Jørgensen, D. V. Muttuvelu, T. Peto, S. Natarajan, J. Davies, P. A. Keane, Lars Holger Ehlers

**Affiliations:** 1 Nordic Institute of Health Economics, Aarhus, Denmark; 2 Department of Ophthalmology, Copenhagen University Hospital, Copenhagen, Denmark; 3 2mitØje ApS, Aarhus, Denmark; 4 Queen’s University Belfast, Belfast, UK; 5 Department of Ophthalmology, Odense University Hospital, Odense, Denmark; 6 Aditya Jyot Eye Hospital Pvt Ltd, Mumbai, Maharashtra India; 7 Global Business School for Health, University College London, London, UK; 8 University College London, London, UK; 9 NIHR Biomedical Research Centre at Moorfields Eye Hospital and UCL Institute of Ophthalmology, London, UK

**Keywords:** Health care economics, Health services

## Abstract

**Background and purpose:**

Optometrist-assisted and teleophthalmology-enabled referral pathway (OTRP) for community optometry referrals has the potential to improve the capacity and efficiency of eye care delivery systems through risk stratification and limiting the number of improved referrals. This study investigates the expected future costs and benefits of implementing OTRP under various possible organizational set-ups relevant to a Danish context.

**Methods:**

A decision-analytic model (decision tree) with a one-year time horizon was constructed to portray alternative future patient referral pathways for people examined in optometry stores for suspected ocular posterior segment eye disease. The main outcomes were total healthcare costs per patient, average waiting time from eye examination in store until the start of treatment or end of referral pathway, and quality-adjusted life-years (QALY) gained. The economic evaluation compares the general ophthalmologist referral pathway (GO-RP) with a potential reimbursement model for the optometrist-assisted teleophthalmology referral pathways (R-OTRP) and a procurement model for the optometrist-assisted teleophthalmology referral pathways (P-OTRP).

**Results:**

The cost per individual with suspected ocular posterior segment eye disease was estimated to be £116 for GO-RP and £75 and £94 for P-OTRP and R-OTRP respectively. The average waiting time for diagnosis or end of referral pathway was 25 weeks for GO-RP and 5.8 and 5.7 for P-OTPR and R-OTPR respectively. QALY gain was 0.15 for P-OTRP/R-OTRP compared to 0.06 for GO-RP.

**Conclusion:**

OTRP is effective in reducing unnecessary referrals and waiting times, increasing patients’ HRQoL, and decreasing the costs of diagnosing individuals with suspected ocular posterior segment eye disease.

## Introduction

The shortage of specialized healthcare providers is a worldwide public health challenge threatening to become a crisis [1, 2]. The ageing population, alarming rise in the prevalence of degenerative disease, and rapid technological innovation are among the factors that increasingly raise the need for healthcare specialists [3, 4]. Ophthalmology is one of the medical specialties with the highest expected future rise in demand for healthcare services, with age-related macular degeneration (AMD), cataracts, glaucoma, and diabetic retinopathy among the most often referred eye diseases [5]. Although the global ophthalmological workforce is growing, the distribution and capacity of the eye care delivery system are universally challenged [6–8]. In most countries, there is a fast-growing need to increase the number of training posts in ophthalmology and ongoing education and training for existing ophthalmologists. As the demand for eye care services continues to grow, it is also essential to explore other innovative solutions to increase capacity and to ensure future patients’ access to timely and high-quality eye care [8–10].

Optometrist-assisted and teleophthalmology-enabled referral pathway (OTRP) for community optometry referrals has the potential to improve the capacity and efficiency of eye care delivery systems through risk stratification and limiting the number of improved referrals [6, 11]. OTRP can be defined as a collaboration between community optometrists and ophthalmologists who are working in either the primary (gate-keep function) or the secondary sector (hospitals), where the community-based optometrist obtains images (e.g., OCT, slit-lamp, or retinal imaging) and transmits them via an electronic system to the ophthalmologist who decides on the case management [11, 12].

One of the primary benefits of OTRP is its potential to increase the capacity of the eye care delivery system by enabling optometrists to play a more significant role in providing comprehensive eye care services. Optometrists are often the first point of contact for patients with eye problems, and they are trained to perform a range of eye exams and diagnose common eye conditions [13]. By collaborating with ophthalmologists, optometrists can provide more comprehensive eye care services, potentially reducing the burden on ophthalmologists and increasing access to eye care for patients. OTRP also has the potential to improve the efficiency of the eye care delivery system by reducing the need for face-to-face consultations between patients and ophthalmologists [12, 14, 15]. This can save patients’ time and money and reduce ophthalmologists’ workloads, allowing them to focus on the most complex cases [12, 15].

From a global perspective, the role of optometrists in national healthcare systems varies between countries, and future OTRP systems will likely differ accordingly [6]. In the United Kingdom (UK), community optometrists conduct nearly all primary eye care consultations, with over 70% funded by the National Health Service [16]. A recent study has demonstrated that more than a third of optometric referrals within the National Health Service did not require specialist consultancy [15] and that OTRP offers the potential for cost reductions and increasing effectiveness [6, 17]. In Denmark, optometrists are not part of the public healthcare system, although they are recognized as healthcare providers [11]. OTRP could potentially play a larger role in the delivery of eye care services in Denmark because optometry stores are widespread across the country, easily accessible to most people, and increasingly integrating automated equipment and diagnostic devices to enhance the accuracy and speed of eye examinations [10].

Despite optometrists being an underutilized resource in the field of eye care in most healthcare systems, no health economic evaluation of OTRP has yet been conducted either in an international or a Danish setting [17]. Therefore, our study aims to investigate the expected future costs and benefits of implementing OTRP under various possible organizational set-ups relevant to a Danish context. This study is designed to inform decision-makers about the possible role of optometrists and teleophthalmology in the national eye care system.

## Materials and methods

### Danish eye care system

The Danish healthcare system is universal and based on principles of free and equal access to healthcare for all citizens [18]. General ophthalmologists maintain a gatekeeper function to the secondary sector (general eye departments or university eye clinics). Danish citizens have the right to schedule an appointment with general ophthalmologists independently, with or without a referral from a general practitioner or optometrist [19]. There are currently 430 ophthalmologists in Denmark, of whom 180 are general ophthalmologists and 250 are employed in the hospital sector [8, 20]. With approximately 5.9 million inhabitants in Denmark, this corresponds to 0.7 ophthalmologists per 10,000 inhabitants, which is a little below the European average of 0.8 per 10,000 inhabitants [21]. General ophthalmologists provide care for approximately 3800 unique patients annually [22], a number that has grown over the last 15 years, especially in rural areas, where waiting times are highest [23]. According to the Danish Health Agency, the number of ophthalmologists must be increased by 40–60% over the next 20 years to maintain current service levels [8].

The density of optometry stores in Denmark is among the highest in Europe and it is approximately three per 10,000 inhabitants [24].

### Organization of a future OTRP system

Two organizational models are particularly relevant for integrating OTRP services in the Danish public healthcare system: a reimbursement (R-OTRP) model and a public procurement (P-OTRP) model.

A reimbursement model is a common way of integrating general ophthalmologists and other private healthcare specialists in the Danish primary care sector. It could be extended to include both optometrists and teleophthalmologists [25]. It is the model currently used for optometrists in many UK National Health Service trusts and for reimbursing private providers under Medicare or Medicaid in the USA. In Denmark, medical specialists and other healthcare professionals can apply for authorization and permission to work under the Danish Health Insurance Act [26]. These professionals can purchase a provider license which gives them the right to practice within a specific geographic domain and up to a certain capacity (or annual cost level) determined by the regional health authority. After receiving the license, the regional health authority is required to compensate for the services provided to patients in accordance with the nationally agreed contractual terms, which include a fee-for-service schedule. The nationally agreed terms of the contract are determined through negotiations every two years between the relevant specialist organization and the public payers. The provider license is typically open-ended with periodic reviews. An advantage of this model is the life-long relationship between payer and provider that enables monitoring and learning. This health economic evaluation assumes that the R-OTRP model is extended to optometrists and teleophthalmologists. We assume that Danish optometrists under an R-OTRP model can achieve the same level of efficiency as UK optometrists through continuous learning and control [12, 14, 15, 27]. We also assume that both optometrists and teleophthalmologists will receive a tariff for their referrals.

A public procurement or tender model is an alternative model used by Danish health authorities. This model is used regularly by Danish health authorities to buy additional capacity for cataract surgery among private ophthalmologists with or without provider licenses [27, 28]. It is also used to procure ambulance services in each of the five regions through competitive bidding between invited private and public service providers for four-year contracts [29] and it is used to create analog competition for hospital pharmaceuticals [30]. The main advantage of a public procurement model is the possibility of price reductions and financial savings on public healthcare budgets through market competition and the flexibility to adjust healthcare capacity to meet temporary fluctuations in demand [31]. In Denmark, the procurement model can be used at the national or regional level following the Danish Public Procurement Law [32] and Procurement Directives from the EU Commission [33]. The duration of the procurement contracts is typically a fixed period, such as one to four years, and winners may be paid for services in different ways according to specific contractual terms. In this economic evaluation, we assume that competitive tenders could be attractive for various partnerships between optometrists and ophthalmologists e.g., optometrists in stores working together with private ophthalmologists (with or without reimbursement contracts with Danish regions), optometrists working with ophthalmologists in hospitals, and general ophthalmologists who employ optometrists. We assume that the P-OTRP model is likely to be cheaper than the R-OTRP model due to price competition, but that the quality of the eye examinations in stores may be higher in the R-OTRP model because of the continuous working relationship between healthcare providers and the optometrist. For simplicity, we further assume that there is only a single fee paid per referred patient under the P-OTRP model covering services performed by a teleophthalmologist and an optometrist.

### Decision-analytic model

**Fig. 1:**
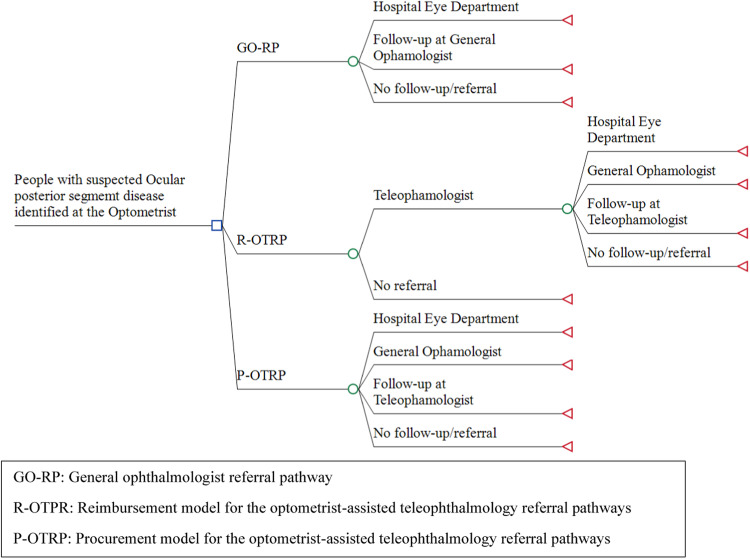
The analytical decision model compares GO-RP vs. R-OTRP vs. P-OTRP. GO-RP: general ophthalmologist referral pathway, R-OTRP: reimbursement model for the optometrist-assisted teleophthalmology referral pathways, P-OTRP: procurement model for the optometrist-assisted teleophthalmology referral pathways, OTRP: optometrist-assisted teleophthalmology referral pathway. For all arms in the model, people with suspected ocular posterior segment eye disease were expected to have their initial comprehensive eye examination in the optometry store. Under GO-RP, patients are referred directly to an ophthalmologist based on the results of the eye exam. Under P-OTRP, the results of the comprehensive eye exam are forwarded digitally to the teleophthalmology service who provides the optometrist with a description of the clinical findings and provides a follow-up/referral plan for each patient, and refers patients to specialized eye care providers. Under R-OTRP, the optometrists are assumed to be able to reduce the number of referrals to the teleophthalmologist compared to P-OTRP (hence the extra branch “no referral”).

A decision-analytic model (a decision tree) with a one-year time horizon was constructed to portray alternative future patient referral pathways for people examined in optometry stores for suspected ocular posterior segment eye disease. The model starts with people having a comprehensive eye examination in an optometry store and ends with the start of treatment or the end of the referral pathway. The model compares three alternative patient referral pathways (Fig. 1): (1) the usual general ophthalmologist referral pathway (GO-RP), where optometrists are not reimbursed by the regional health authorities for the eye examination but refer all patients without any involvement of a teleophthalmologist to a general ophthalmologist, (2) an R-OTRP model, and (3) a P-OTRP model, as described in section 2.2.

The economic evaluation was conducted from a Danish public health sector perspective with the main outputs being total healthcare costs per patient, average waiting time from eye examination in store until the start of treatment or end of referral pathway, and quality-adjusted life-years (QALY) gained. The QALYs were calculated as the difference between the gain in health-related quality of life (HRQoL) from initiation of treatment minus any disutility from potential anxiety during waiting time. As a sensitivity analysis, we included a societal perspective to explore the consequences for patients in terms of transportation and productivity costs. The model was constructed using TreeAge Pro Healthcare (version 2022, R2.0) following international guidelines for health economic evaluation [34, 35].

### Model inputs

The model was parameterized using the best available evidence relevant to the model (Table 1). Central model assumptions were validated using an independent expert panel, comprising three general ophthalmologists, two optometrists, and one associate professor of health economics.

**Table 1:** Base-case inputs to the decision-analytic model.

Variable	Base-case	Sources
*Cohort characteristics*		
Share of cohort with ocular segment posterior disease	0.195	Muttuvelu et al. [11], Kern et al. [12]
Type of eye disease among patients with ocular posterior segment disease	AMD (≈20–21% of patients at the general ophthalmologist), glaucoma and glaucoma screening (≈20–27%), cataract (≈9–15%), retinopathy (≈25–33%), and other ocular posterior segment disease (≈10–20%).	Expert panel
Share of cohort referred to hospital eye department	0.012	Muttuvelu et al. [11]
Share of cohort referred to general ophthalmologist with OTRP	0.183	Muttuvelu et al. [11]
*Probabilities*		
Share of cohort that consult general ophthalmologist after referral from optometrist (GO-RP)	0.9	Expert panel
Share of patients referred from optometrist that teleophthalmologist do not refer to general ophthalmologist	0.805	Muttuvelu et al. [11]
Share of referrals to teleophthalmologist reduced using R-OTRP compared to P-OTRP	0.1	Expert panel
Share of teleophthalmologist patients referred to follow-up at teleophthalmologist	0.661	Muttuvelu et al. [11]
Share of teleophthalmologist patients with no further referral or follow-up	0.144	Muttuvelu et al. [11]
*Cost (2022, £)*		
Average first visit at general ophthalmologist	101	Danish ophthalmologists collective agreement [37] Supplementary Table A1
Follow-up consultation general ophthalmologist	101	Danish ophthalmologists collective agreement [37]
Hospital eye department	127	Danish DRG tariffs 02MA01 [38]
Tariff for teleophthalmologist	46	Expert panel
Tariff for optometrist	20	Expert panel
*Waiting time (weeks)*		
General ophthalmologist	27.5	Sundhed.dk [20]
Hospital eye department (after general ophthalmologist)	26.0	Sundhed.dk [20]
Acute/urgent referrals from optometrist to teleophthalmologist	0.14	Muttuvelu et al. [11]
Non-acute/routine referrals from optometrist to teleophthalmologist	0.43	Muttuvelu et al. [11]
*Utility/disutility*		
Anxiety (disutility in false positives)	0.02	Expert panel
Treatment effect	0.2	Expert panel

*AMD* age-related macular degeneration, *QALY* quality-adjusted life-years, *GO-RP* general ophthalmologist referral practice, *R-OTRP* reimbursement model for optometrist-assisted teleophthalmology referral pathway, *P-OTRP* procurement model for optometrist-assisted teleophthalmology referral pathway.

The assumptions about cohort disease prevalence were taken from Muttuvelu et al. [11]. We assume the same share of patients with eye disease and the same share of patients referred to treatment at a hospital eye department and general ophthalmologist for all three alternatives (GO-RP, R-OTRP, and P-OTRP) i.e., the clinical quality is assumed not to be affected by the introduction of teleophthalmologist and choice of organizational form.

In the base-case, we assume that all patients in GO-RP see a general ophthalmologist if an optometrist gives the patient a diagnosis after a comprehensive eye examination, but in the sensitivity analyses, this assumption is relieved down to 50%. In base-case analysis for P-OTRP, we assume that teleophthalmologist can reduce the number of referrals up to 80.5% [11], which is varied in the sensitivity analysis from 50–90%. In base-case of R-OTRP, we assume that optometrists can reduce the number of referrals to teleophthalmologist by 10% compared to P-OTRP, which is increased in the sensitivity analysis up to 20%.

All monetary outcomes were estimated in Danish Krone (DKK) adjusted to the year 2022 using the Consumer Price Index [36] and subsequently converted to 2022 British Pound Sterling (£) using a conversion rate on December 12, 2022 of DKK 100 = £11.57. Healthcare costs were obtained from published sources, including the Danish diagnosis-related groups tariff system [37] and tariffs from the Danish ophthalmologists’ collective agreement [38]. The costs/tariffs of teleophthalmologists and optometrists were estimated in base-case to be £46 (DKK 400) and £20 (DKK 175) respectively. The model only includes marginal costs of services from providers, and no attempts have been made to include administrative costs of establishing and running a OTRP system such as the costs of tendering quality assurance or reimbursement. Nor have any potential changes in the costs of implementation been included.

Data on current waiting times in the Danish eye care system were incorporated as average weeks of waiting time for general ophthalmologists and hospital eye departments according to available Danish statistics and validated with the expert panel [20].

QALY gain was included within the one-year horizon as the gain from initiation of treatment of eye disease assuming an increase in HRQoL of 0.2 measured on an EQ-5D scale [39]. The disutility from potential anxiety in the waiting time from eye examination and optometrist’s diagnosis and the start of treatment (for people with confirmed diagnosis) or ophthalmologist diagnosis (false positives) was included, assuming a difference in HRQoL of the average referred patient measured on an EQ-5D scale of 0.02 [39].

Furthermore, the main results are shown graphically in a cost-effectiveness plane constructed from a probabilistic sensitivity analysis with 10,000 2nd-order Monte Carlo simulations using beta distribution for probabilities and QALYs, and gamma distributions for costs and waiting times [40]. In the sensitivity analysis, patients’ transportation costs were included, assuming an average transport cost per consultation at the general ophthalmologist and hospital eye department of £11.75. We further included productivity costs due to patients’ absence from work because of eye consultations, assuming an average cost per consultation at the general ophthalmologist and hospital eye department of £20.83 [34].

## Results

In the base-case analysis, the cost per individual with suspected ocular posterior segment eye disease was £115 for GO-RP and £75 and £94 for P-OTRP and R-OTRP respectively (Table 2). The average waiting time for diagnosis or end of referral pathway was 25 weeks for GO-RP and 5.8 and 5.7 for P-OTPR and R-OTPR respectively.

**Table 2:** Base-case results for estimated costs and consequences (base-case analysis).

Strategy	Cost, £ (CI)	Waiting time, weeks (CI)	Potential QALY gain (CI)
GO-RP	116 (89–146)	25.0 (16.3–35.7)	0.06 (0.04–0.09)
R-OTRP	94 (73–120)	5.7 (3.9–7.9)	0.1523 (0.0963–0.2169)
P-OTRP	75 (54–101)	5.8 (3.5–8.7)	0.1519 (0.0959–0.2169)

*GO-RP* general ophthalmologist referral practice, *R-OTRP* reimbursement model for optometrist-assisted teleophthalmology referral pathway, *P-OTRP* procurement model for optometrist-assisted teleophthalmology referral pathway, *QALY* quality-adjusted life-years.

**Fig. 2:**
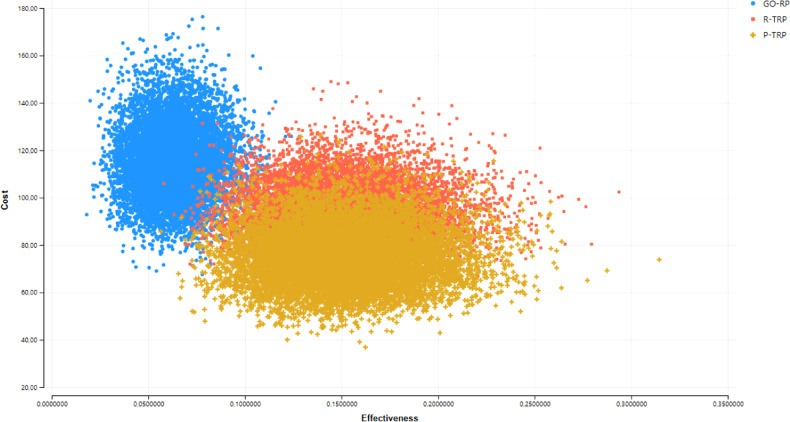
Cost-effectiveness scatterplot. A graphical presentation of expected cost and QALYs per individual with suspected ocular posterior segment eye disease for the different referral pathways.

Both P-OTPR and R-OTPR were associated with a potential QALY gain of approximately 0.15 compared to 0.06 for GO-RP. The cost-effectiveness scatterplot indicated a high probability of OTRP being both less expensive and more effective than GO-RP (Fig. 2). The probabilistic sensitivity analysis showed that P-OTRP was cheaper than GO-RP and R-OTRP in more than 95% of the simulations.

The deterministic analysis demonstrated that the results were sensitive to the assumption about the share of the cohort that consults general ophthalmologists after a referral from an optometrist (GO-RP, base-case = 90%) (Table 3). Furthermore, the result was sensitive to the size of the teleophthalmologist tariff. On the other hand, a potential reduction in the cost of the first visit to the general ophthalmologist did not significantly impact the result; the main reason is that a change in this cost will affect all arms. The sensitivity analyses showed that the results were also influenced by the effectiveness of P-OTRP and R-OTRP in reducing the number of unnecessary referrals but GO-RP would not surpass the OTRPs. The result was not sensitive to changes in the assumptions about zero false positives from teleophthalmologist to general ophthalmologist, however, assuming more than 30% of false positives led to R-OTRP being cheaper than P-OTRP.

**Table 3:** Results of sensitivity analyses.

	Strategy								
	GO-RP			R-OTRP			P-OTRP		
Variable	Cost, £	Waiting time, weeks	Potential QALY gain	Cost, £	Waiting time, weeks	Potential QALY gain	Cost, £	Waiting time, weeks	Potential QALY gain
**Base-case**	**115**	**25.0**	**0.06**	**94**	**5.7**	**0.1523**	**75**	**5.8**	**0.1519**
Cost of first visit at general ophthalmologist (base-case = £101)									
*£80*	96	a	a	71	a	a	50	a	a
*£110*	123	a	a	96	a	a	77	a	a
*£120*	132	a	a	98	a	a	79	a	a
Tariff teleophthalmologist (base-case = £46)									
*£25*	a	a	a	75	a	a	53	a	a
*£75*	a	a	a	126	a	a	110	a	a
*£100*	a	a	a	154	a	a	140	a	a
Tariff optometrist (base-case = £20)									
*£0*	a	a	a	71	a	a	a	a	a
*£10*	a	a	a	83	a	a	a	a	a
*£25*	a	a	a	100	a	a	a	a	a
Share of cohort that consult general ophthalmologist after referral from optometrist (GO-RP) (base-case = 90%)									
50%	64	13.9	0.04	a	a	a	a	a	a
70%	90	19.5	0.05	a	a	a	a	a	a
100%	128	27.8	0.07	a	a	a	a	a	a
Reduction in the number of referrals to general ophthalmologist with P-OTRP compared to GO-TP (base-case = 80.5%)									
*50%*	a	a	a	a	a	a	102	14.2	0.1616
*70%*	a	a	a	a	a	a	85	8.7	0.1553
*90%*	a	a	a	a	a	a	67	3.2	0.1489
Reduction in the number of referrals to teleophthalmologist using R-OTRP compared to P-OTRP (base-case = 10.0%)									
*0%*	a	a	a	99	5.8	0.1523	a	a	a
*5%*	a	a	a	96	5.7	0.1434	a	a	a
*15%*	a	a	a	89	5.7	0.1256	a	a	a
*20%*	a	a	a	86	5.7	0.1167	a	a	a
Societal perspective									
***Full*** ***societal***	197	a	a	112	a	a	93	a	a
*Transport*	10	a	a	2	a	a	3	a	a
*Patient* *cost*	71	a	a	15	a	a	15	a	a

Bold values: base-case represents a health sector perspective. Full societal perspectives adds cost of transportation and patients' time.

*a* No change relative to base-case, *QALY* quality-adjusted life-years, *GO-RP* general ophthalmologist referral pathway, *P-OTRP* procurement model for optometrist-assisted teleophthalmology referral pathway, *P-OTRP* reimbursement model for optometrist-assisted teleophthalmology referral pathway.

When patients’ cost of transportation and productivity costs were included, the OTRP appeared even more cost-effective as OTRP reduces patients’ travel costs and productivity costs compared to GO-RP.

## Discussion

This study is, to our knowledge, the first health economic evaluation of optometrist-assisted teleophthalmology. Based on the best available evidence, the results strongly indicate that the role of OTRP in future eye care delivery systems should be planned for. OTRP has the potential to reduce healthcare costs and waiting time, increase patients’ HRQoL, and decrease patients’ cost of transportation and productivity costs. The main reason for these benefits is the ability of OTRP to alleviate the burden on general ophthalmologists.

The results are sensitive to assumptions about the size of the tariffs for teleophthalmology services and the number of unnecessary referrals in the future eye care system. Furthermore, the conclusion about cost-effectiveness will also depend upon the size of the administrative costs in establishing and running a national OTRP system. These administrative costs could be seen as an investment in a more effective national eye care system which is paid for by a reduction in marginal costs for everyone who receives a comprehensive eye examination in the OTRP setup. Thus, OTRP is more likely to be cost-effective in a large-scale implementation rather than a small-scale intervention. Scalability will, therefore, be an important issue in future OTRP systems. In Denmark, more than 690,000 patients are currently being treated in general ophthalmology practices [22]. Assuming, for example, that 15% of these patients could be seen in a future OTRP system with a similar cost saving of approximately £20–40 per patient, annual marginal cost savings of £2.1 m to £4.1 m (DKK 18.2 m–35.4 m) could be realized.

This study has several limitations. These include uncertainties in the input data for probabilities of referrals for OTRP, costs, and QALYs, and the lack of consideration for individuals’ preferences for patient pathways, which should have been included in a full benefits assessment [41]. The potential risk of false negatives due to optometrists’ and teleophthalmologists’ referral quality and competencies not being as high as general ophthalmologists were not considered. In this study, we assume a high accuracy of remote diagnoses [11]. Although there is a possibility of poor-quality images, advancements in camera technology have proven their efficiency when compared to face-to-face examination and consultation, however, more research on this topic is needed [42, 43]. Additionally, the effects of teleophthalmology on workforce dynamics were not addressed in our calculations.

In the future, AI-powered OTRP is expected to outperform other OTRPs, particularly in terms of accessibility, convenience, and scalability [43]. These aspects were not incorporated in the calculations but would probably have increased the possibilities of future savings from OTRP. The P-OTRP model will have an advantage in terms of scalability because it builds on market competition and standardized products and services rather than education levels and competencies in optometrist stores.

The generalizability of results from health economic evaluations is usually limited due to the differences among countries with regards to the organization of healthcare, clinical practices, unit costs, etc. [41]. Currently, OTRPs are being tested in clinical research at university hospitals in the UK [44]. For research and quality assurance purposes, both centralized private teleophthalmology units and university hospitals involved in OTRP have an important role in data collection, research, and continuous quality improvement. The P-OTRP model can involve many types of providers including ophthalmologists who are working in public as well as private organizations. Market competition secures the economic advantages of this particular model.

The use of OTRP will require a secured digital communication system between the optometrist and the ophthalmologist. In Denmark, such systems are already in place and enforce the Danish Data Protection Act and the European General Data Protection Regulation [45–47]. Therefore, implementation of the OTRP in Denmark will be a marginal cost in relation to the already established systems. However, this may not be generalizable to other countries with other prerequisites for establishing secure communication systems.

## Conclusions

Optometrist-assisted teleophthalmology is effective in reducing unnecessary referrals and waiting times, increasing patients’ HRQoL, and decreasing the healthcare and societal costs of diagnosing individuals with suspected ocular posterior segment eye disease. Further empirical research is needed to investigate the potential for improvements in national eye care through optometrist-assisted teleophthalmology.

## Summary

### What was known before:

- Teleophthalmology represents an effective means for triaging patients; however, the cost-effectiveness of such services remains unexplored in the scientific literature.

### What this study adds:

- This research represents the first health economic evaluation of a nationwide teleophthalmology service, aiming to quantify potential economic savings, gains in Quality-Adjusted Life-Years (QALY), and reductions in waiting times.

## Data Availability

All data generated or analyzed during this study are included in this published article. The TreeAge model is available from the corresponding author on reasonable request.
